# Effectiveness of Mulligan manual therapy over exercise on headache frequency, intensity and disability for patients with migraine, tension-type headache and cervicogenic headache – a protocol of a pragmatic randomized controlled trial

**DOI:** 10.1186/s12891-021-04105-y

**Published:** 2021-03-03

**Authors:** Kiran Satpute, Nilima Bedekar, Toby Hall

**Affiliations:** 1 Department of Musculoskeletal Physiotherapy, Smt. Kashibai Navale College of Physiotherapy, Off Westerly by Pass, Narhe, Pune, Maharashatra 411041 India; 2 Department of Musculoskeletal Physiotherapy, Sancheti Institute College of Physiotherapy, Thube Park, Shivaji Nagar, Pune, Maharashatra 411001 India; 3 School of Physiotherapy and Exercise Science, Curtin University, P.O. Box U1987, Perth, WA 6845 Australia

**Keywords:** Headache, Migraine, Tension-type headache, Cervicogenic headache, Manual therapy, Mulligan manual therapy, Disability

## Abstract

**Background:**

Non - pharmacological management of migraine, tension-type headache (TTH), and cervicogenic headache (CGH) may include spinal manual therapy and exercise. Mulligan Manual Therapy (MMT) utilizes a protocol of headache elimination procedures to manage headache parameters and associated disability, but has only been evaluated in CGH. There is little evidence for its effectiveness in migraine and TTH. This study aims to determine the effectiveness of MMT and exercise over exercise and placebo in the management of migraine, TTH, and CGH.

**Methods:**

This pragmatic trial is designed as a prospective, three-armed randomised controlled trial in a clinical setting provided at a general hospital physiotherapy department. Two hundred ninety-seven participants with a diagnosis of migraine, TTH or CGH based on published headache classification guidelines will be included. An assessor blind to group allocation will measure outcomes pre-and post-intervention as well as 3 and 6 months after commencement of treatment. Participants will be allocated to one of the three groups: MMT and exercise; placebo and exercise; and exercise alone. The primary outcome measure is headache frequency. Secondary outcome measures are headache duration and intensity, medication intake, pressure pain threshold (PPT), range of motion recorded with the flexion rotation test, and headache disability recorded with Headache Activities of Daily Living Index (HADLI). The intention-to-treat principle will be followed for statistical analysis. Between groups differences for all outcome measures at baseline and at reassessment points and 95% confidence intervals will be calculated using a mixed model ANOVA. Post hoc tests will be conducted to identify any significant difference between groups and over time.

**Discussion:**

This pragmatic study will provide evidence for the effectiveness of MMT when compared with a placebo intervention and exercise on headache frequency, intensity, and disability. Limitations are that baseline evaluation of headache parameters may be affected by recall bias. External validity will be limited to the population with a minimum 1-year history of headache. The HADLI is not yet extensively evaluated for its psychometric properties and association between PPT and headache parameters is lacking. Performance bias is inevitable as a single therapist will be delivering all interventions.

**Trial registration:**

The trial was registered prospectively under the Clinical Trial Registry India (Registration number: CTRI/2019/06/019506, dated on 03/06/2019). .

## Background

Headache is one of the most common disorders globally, with a potential for major disability. Migraine, tension-type headache (TTH), and cervicogenic headache (CGH) are common types of headache which negatively impact on quality of life, work activities, and family life posing a direct or indirect economic burden on society [1]. The global 1-year prevalence of primary headache in adults is 47% [2] with a lower but still substantial prevalence for specific headache disorders. The 1-year prevalence for migraine, TTH, and CGH in adults was documented as 15%, 21% [3], and 4% respectively [4].

Even though well defined in the third edition of the International Classification for Headache Disorders (ICHD) 2018 [5], headache diagnosis can be difficult due to the overlap of symptoms between migraine, TTH, and CGH [6, 7]. Moreover, in clinical practice, multiple headache forms may co-exist in up to 55% of cases [8]. This may explain the uncertainty in the initial diagnosis and subsequent shift in headache categorization that occurs in 40% of cases at subsequent follow-up [9]. Thus it is important to identify the predominant type of headache before planning management, for optimum patient care [10] .

The non-pharmacological management of common headache types can include physiotherapy [11]. This may consist of exercise and spinal manual therapy, the latter of which is often provided due to the presence of neck pain in these patients [12]. Neck pain is characteristic of CGH [5] but is also very common in people who suffer from migraine and TTH [13]. In migraine, neck pain is more prevalent than nausea [14] and is positively associated with headache frequency and increases overall headache related disability [15, 16].

It has been thought that neck pain occurring with headache could be due to cervical articular impairment [16, 17] or poor motor control of neck muscles [18, 19]. In contrast, neck pain may also be a symptom of headache which may be unrelated to musculoskeletal issues [20]. Various physical examination tests have been described to identify musculoskeletal dysfunction in headache which can guide non-pharmacological management [21]. However, the evidence regarding the importance of these clinical tests and therefore musculoskeletal dysfunction in headache diagnosis is poor [22].

Various systematic reviews favor the use of manual therapy as a part of non-pharmacological management of migraine [23, 24], TTH [25, 26], and CGH [27, 28]. It has been thought that manual therapy may modify identified articular dysfunction, particularly in the upper cervical spine, as well as improve muscle function and motor control.

In addition to postulated biomechanical effects, manual therapy has been shown to reduce the sensitivity of the trigeminocervical nucleus which is known to be a factor in headache pathophysiology [29]. For example, headache reproduction and resolution following manual palpation of the upper cervical spine in patients with primary headache [29, 30] indicates that manual therapy has the capacity to modulate the sensitivity of this nucleus.

Sensitization of the trigeminocervical nucleus appears to be a common feature in migraine, TTH and CGH [31]. Although such sensitization may have arisen through different means in each of the headache forms, desensitization of this nucleus by manual therapy and exercise may theoretically be a viable treatment option [12].

Mulligan manual therapy (MMT) is a relatively new concept that utilises pain-free low-velocity joint mobilisation techniques that can include an active movement component [32]. In this concept, pain-free sustained manual force is applied to the upper cervical spine in an attempt to modify headache or increase upper cervical spine mobility. If successful the technique becomes the treatment. If not then a new technique is trialled until all techniques are exhausted. The MMT protocol in headache management is essentially a symptom and impairment elimination approach and is indicated only if a substantial headache reduction and/or improvement in range of motion occurs as a result of the applied technique. Previous research of MMT has focused mainly on CGH [33]. The effects of such an approach on other headache forms have been reported in a case study [34] of a patient with features of migraine, but not in TTH and not in a formal RCT. Thus there is scope to explore the comparative effectiveness of MMT in the management of CGH, migraine, and TTH.

This paper aims to report the study protocol which will be used to investigate the short and mid-term effectiveness of MMT and exercise on headache frequency, intensity, disability, and duration as well as medication intake, upper cervical rotation range of motion, PPT and patient satisfaction compared with placebo and exercise and exercise alone in the management of migraine, TTH, and CGH. The objective is to investigate whether MMT with exercise is more effective than placebo MMT with exercise or exercise alone. Our primary hypothesis is that there will be a 50% reduction in headache frequency in the experimental group (MMT with exercise) and recovery is greater than that seen in the 2 control groups (placebo MMT with exercise and exercise alone).

## Methods/ design

Approval and registration of the study is confirmed. The design of this clinical trial follows the recommendations of the SPIRIT guidelines (2013) [35].

**Fig. 1:**
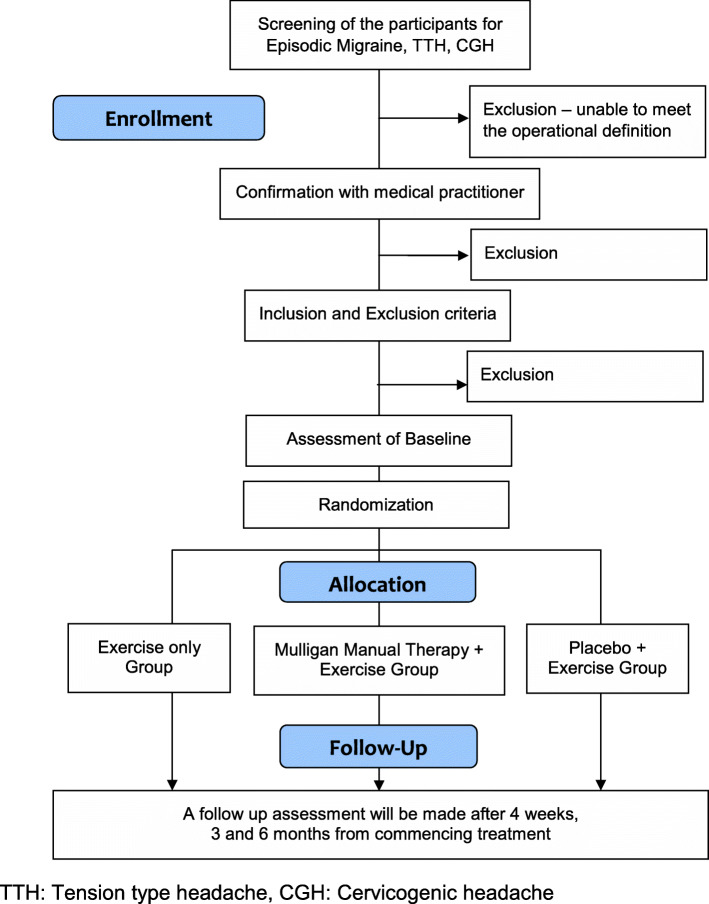
Flow chart of the study process based on SPIRIT guidelines (2013)

This is an assessor blind pragmatic randomized clinical trial with three parallel groups (MMT + exercise, placebo + exercise, and exercise alone) based on SPIRIT guide lines (2013) [35]. A chart indicating the flow of participants through the study is shown in Fig. 1.

The study will be conducted at the physiotherapy department of Smt. Kashibai Navale Medical College and General Hospital, Maharashtra, India. This hospital is a multispecialty general hospital. Ethical approval was obtained from the ethical committee of the Smt. Kashibai Navale College of Physiotherapy (Approval number: SKNCOPT/IEC/2019/208) and institutional review board of Sancheti Institute for Orthopedic and Rehabilitation, Pune (Approval number: IRB-SIOR/ Agenda 049). Both committees are responsible for ethics and data monitoring throughout the trial. The trial was registered prospectively under the Clinical Trial Registry India (Registration number: CTRI/2019/06/019506, dated 03/06/2019).

### Eligibility criteria

Consecutive participants with episodic migraine, episodic TTH and CGH referred to the physiotherapy outpatient department by the hospital medical department will be recruited into this trial. A diagnosis of episodic migraine, episodic TTH and CGH will be made by the primary investigator under the guidance of a medical practitioner as per the ICHD -3 [5]. Episodic TTH is at least 10 episodes of headache occurring on 1–14 days/month on average for > 3 months (≥12 and < 180 days/year) and lasting from 30 min to 7 days. Episodic migraine is that which occurs on less than 15 days per month for at least 3 months [5].

Participants will be included if they fulfil the following inclusion criteria: age more than 18 years and less than 60 years, pain intensity > 6 on a 10 cm visual analogue scale at the time of presentation (to balance headache severity across different headache groups), a minimum 1-year history of headache with a minimum mean frequency of 1 per week. This frequency was chosen to enable effective monitoring of outcome, in particular with respect to treatment intervention. Less frequent headache would make it difficult to monitor progress. An additional criterion is hypomobility of the upper cervical spine (C0–3) on manual examination. Manual examination has been shown to be reliable when used in the upper cervical spine [36], and has been used in previous studies exploring the effectiveness of manual therapy for headache [37–40]. By way of standardization, an upper cervical spine joint is classified as impaired if moderate or marked tissue resistance is perceived along with local or referred pain of > 2 on a 0–10 cm visual analogue scale on palpation [41]. A further criterion is the reproduction of headache on palpation of the upper cervical spine (C0–3) [29].

Participants will be excluded if their headache diagnosis is other than, migraine, TTH or CGH. In addition, exclusion criteria are instability of the upper cervical spine, evidence of cervical arterial insufficiency observed during clinical testing, history of vertigo or dizziness, rheumatoid arthritis, ankylosing spondylosis, cervical spine fractures, pregnancy, cognitive compromise, and any other contraindications to manual therapy.

### Procedures

Participants will be provided with an information sheet outlining the study protocol including duration of commitment, intervention, benefits and harms of the treatment, voluntary participation, right to withdraw, as well as confidentiality of data. It will be explained that no compensation will be given if any adverse event occurs and study findings may be published but without revealing participants identity.

Those willing to participate will be enrolled and asked to provide signed written informed consent with the right to withdraw at any time. After recruitment, at initial assessment, a qualified therapist will explain the study procedures and collect demographic data. Confidentiality will be maintained throughout the trial.

Participants will then be randomly and equally allocated to one of three groups: MMT plus exercise; placebo plus exercise; or exercise alone by stratified randomization based on the type of headache. Randomization will be achieved using a computer-generated sequence received in advance from an independent statistician and will be hidden in sequentially numbered opaque sealed envelopes by a research assistant. All participants will be asked to not reveal their group identity and are advised to continue with their regular medical management. Any change in the protocol will be communicated with the ethics committee and if approved appropriate changes will be made in the Clinical Trial Registry.

### Interventions

Each subject will receive 6 treatment sessions spread over 4 consecutive weeks and each treatment session will not extend beyond 30 min. A principal investigator will perform all the interventions. The principal investigator is a physiotherapist with a Masters degree in musculoskeletal physiotherapy with 15 years of clinical experience who has undertaken the highest level of training in MMT.

All participants will be informed that their designated intervention has been shown to improve headache symptoms. Treatment will cease if the subject withdraws consent, has an increase in pain or discomfort due to the treatment, or develops any contra-indication during the intervention period. Additionally subjects will be withdrawn if they receive any other physiotherapy program including massage, manual therapy or chiropractic treatment to the neck or shoulder region. All participants will be requested to avoid any forms of manual therapy or massage until the final evaluation at 6 months.

All participants will receive the following structured exercise programme encompassing conventional cervical flexion loading exercise [42], upper quarter low load endurance training, stretching, and generalized mobility exercises [43]. All exercises will be supervised during each session and exercise parameters will be adjusted if required but without any modifications in the type of exercise.

Exercises will be performed in the following sequence. 1. A conventional cervical flexion loading exercise [42] is performed with the subject in a supine position with the knees bent and neutral head/neck position. The head lift exercise will be taught ensuring that the craniocervical spine will be maintained in a neutral position while lifting the head from the supporting surface [42]. 2. Scapular retraction in prone with the subjects arms by their side, performed against gravity resistance. For the first 2 weeks, 2 sets of 5–10 repetitions with a 10 s hold of the above two exercises will be performed, building slowly to 3 sets of 15 repetitions with 10–15 s hold over the next 4 weeks. 3. Passive static self-stretching exercises for the upper trapezius, levator scapulae, scalene, and sternocleidomastoid muscles will be delivered only if tightness is perceived on assessment. Stretching will be maintained for 30 s with 3 repetitions given to each tight muscle. 4. Active mobility exercises of the neck for flexion, extension, side flexion and rotation to either side will be included [29]. Two sets of 10 repetitions will be performed. Participants will be advised to undertake similar exercise at home, unsupervised, once a day. Participants will be asked to maintain an exercise diary to monitor compliance.

**Fig. 2:**
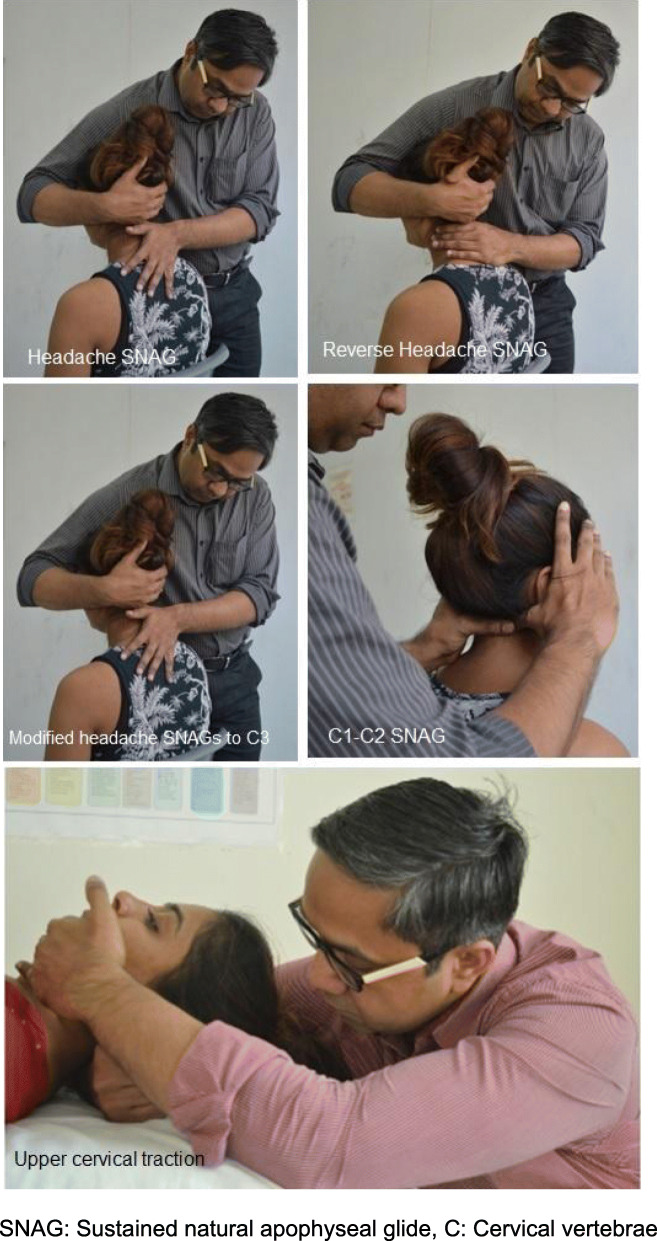
Mulligan Manual Therapy techniques

The group receiving MMT and exercise in addition to the exercise program will receive the MMT protocol for headache. MMT will be delivered at the discretion of the therapist, based on the initial and progressive assessment of the participant’s cervical joint dysfunction and headache presentation. The protocol is as follows (Fig. 2):

#### Technique 1

Headache SNAG: A posteroanterior mobilisation of the second cervical vertebrae is sustained for 10 to 30 s with the aim to reduce headache intensity at the time of application. A maximum of 6 repetitions will be given if there is a reduction in headache at the time of the first application [44].

#### Technique 2

Reverse Headache SNAG: In the event of a failed response in terms of pain reduction to a Headache SNAG, an anteroposterior mobilisation of the second cervical vertebrae is sustained for 10 to 30 s depending on response. A maximum of 6 repetitions will be given if there is a reduction in headache intensity at the time of the first application [44]. Both of the above interventions will be delivered with the patient in a comfortable sitting position while the therapist cradles the participant’s head with one hand while the other hand applying the mobilisation force [44].

#### Technique 3

Modified headache SNAG to C3: In the event of a failed response to a Headache SNAG, and Reverse Headache SNAG, a posteroanterior mobilisation of the third cervical vertebrae is sustained for 10 to 30 s depending on response. A maximum of 6 repetitions of the successful technique will be given if there is a reduction in headache at the time of the first application [34].

#### Technique 4

Upper cervical traction: In the event of a failed response in terms of pain reduction to the previous techniques, upper cervical traction will be delivered with the participant in a supine position. The therapist pronates their forearm against the subject’s occiput while fixing the subject’s chin. The resultant traction force will be sustained for 10–30 s with the aim to reduce headache intensity. A maximum of 10 repetitions will be delivered in a single session [44].

#### Technique 5

C1-C2 SNAG: If the subject does not present with a headache at the time of the assessment, the flexion-rotation test (FRT) [45] will be evaluated. If a 10° restriction is identified compared to the reference standard of 44°, a unilateral posteroanterior mobilisation will be applied to the transverse process of the first cervical vertebrae on the contralateral side of restriction on the FRT. The subject will be asked to rotate the head towards the restricted side of the flexion-rotation test as far as they are able without pain. A maximum of 3 repetitions will be applied in a single treatment session. If impairment is not identified on the FRT and there is no headache symptoms present, then only exercise will be provided as there is no further option for treatment using MMT.

The third group will receive a placebo replicating the Headache SNAG technique but without any manual force applied. The position will be held for 10 to 30 s and 6 repetitions applied. The duration of the placebo treatment session will be similar to that in the MMT group. Participants will continue to receive the same exercise protocol as the other groups. Any increase in headache intensity or neck pain for more than 1 h or dizziness during or after the intervention will be documented as an adverse event. In such cases, participants will be referred to a medical practitioner of the hospital and withdrawn from the study.

### Outcomes

Outcome measures will be taken at base line just after enrolment, as well as at 4 weeks, 3 months and 6 months following treatment commencement by an assessor, blind to group allocation and intervention. To assess whether the participants were blind to intervention or not, after each treatment session the participants in the MMT and the placebo group will complete a questionnaire on whether they believed MMT treatment was received, partly received or not.

The primary outcome measure is headache frequency [46] which is the total number of headache days per month noted in a headache diary. Secondary outcome measures are as follows: headache intensity [47] recorded on a 10 cm visual analogue scale, which is considered a valid and reliable tool for measuring pain intensity [47]. Headache duration [46] will be measured in hours per week. Headache frequency and duration are considered a valid method of measurement in the headache population [46]. Medication Intake will be recorded as total tablets consumed per week. PPT will be measured using a digital algometer with a surface area of 1 cm kg/cm^2^, with pressure applied at the rate of 1 kg/ cm^2^ /s perpendicular to the skin. PPT will be assessed over the suboccipital muscles, C2–3 zygapophyseal joint and upper trapezius muscle bilaterally [48] to assess local hypersensitivity, and over the tibialis anterior muscle to assess central sensitisation. Reliability for measuring PPT was reported as excellent (ICC: 0.82–0.99) [49]. Headache disability will be recorded with the Headache Activities of Daily Living Index (HADLI) [50] which is a 9 item self-reported questionnaire designed to measure the impact of headache on quality of life. Each item has 6 sub-questions which are rated from 0 to 5 thus the maximum score of 45 indicates a severe disability. Patient satisfaction will be assessed using a 0–100% numeric rating scale. Passive upper cervical rotation range in degrees to both sides will be measured with the FRT using a previously reported reliable and valid method [45]. A compass application on a smart phone mounted on the patient’s head with Velcro straps will be used to measure range of motion. Reliability of the FRT was reported as good to excellent in subjects with migraine (0.71–0.78) and CGH (ICC: 0.73–0.83) [51].

### Statistical methods

The sample size for this study was calculated based on the primary outcome of headache frequency. According to the International Headache Society guidelines (2018), a 50% reduction in headache frequency is considered a clinically significant difference. A sample size of 297 participants will be required for the study based on a priori power analysis with a power of 0.8 and an alpha of 0.05 assuming 10% drop-outs.

All data entry will be carried out by research assistants who will be blind to treatment allocation. The final trial dataset will be accessible to all investigators and a statistician. Data will be stored with the principal investigator. Descriptive statistics will be performed to examine baseline parameters and demographic data. Demographic data will include age, gender, symptom duration, occupation, height, and weight. Baseline parameters will include the headache frequency, intensity, duration, medication intake, PPT, HADLI score, and FRT range of motion. Parametric tests will be used for normal distributions and ratio level data. Between groups differences for the primary and secondary outcome measures at baseline and at reassessment points and 95% confidence intervals will be calculated using mixed ANOVA. Post hoc tests will be conducted to identify any significant difference between groups and over the time. The intention-to-treat principle will be used if loss of follow up is not more than 10%. Non-parametric tests will be applied for non-normal distributions and ordinal level data.

## Discussion

To our knowledge, this will be the first pragmatic RCT to evaluate the effects of MMT in the management of migraine, TTH and CGH. A pragmatic effectiveness study design will enable a greater understanding of the true effect of MMT when compared with a placebo intervention and a control group in reducing various parameters of headache over a 6 months period. A placebo effect is associated with all physiotherapy interventions however its contribution may vary. This study will help us to understand the true net effect of MMT over placebo.

Recruitment will be a challenge due to the strict inclusion criteria but that could be strength of our study to involve a homogeneous population of headache sufferers with features of upper cervical articular dysfunction who might respond to MMT.

Previous studies using MMT targeting the upper cervical spine for headache management have not reported adverse events associated with its application. Thus this approach appears to be safe for its clinical use [52]. All participants will continue to take their medications as prescribed by their medical practitioner.

Several limitations of the study protocol must be discussed. Headache differentiation into one of three categories will be challenging due to the overlap of features among headache forms. The medical practitioner’s experience in differential diagnosis may help to overcome this. Baseline evaluation of headache parameters may be affected by recall bias, this is inevitable when recalling headache, but would be the same for each headache group. A further issue is that generalisation of results may only be limited to the headache population with a minimum 1-year history of headache. Headache is a chronic disease and the use of non-pharmacological management in those with headache for less than 1-year is not common practice in our hospital facility. All treatments will be delivered by one therapist possibly creating a performance bias. However, the therapist will ensure that each intervention is applied with equal emphasis and confidence. In addition, all subjects will be asked if they received the intervention which may provide a measure of performance bias. The home exercise programme is unsupervised but compliance will be evaluated with an exercise diary. The HADLI questionnaire is not yet extensively evaluated for its psychometric properties but studies are currently underway to evaluate those properties. Lastly, the outcome measure PPT lacks standardization, cannot be used in diagnosis or screening [53], and has no association with other headache parameters [49]. However, PPT is included to assess local and peripheral hypersensitivity which is one of the common features of central sensitisation [54]. The effect of MMT on local and peripheral hypersensitivity has been measured indirectly with PPT in other peripheral musculoskeletal conditions, for example at the knee joint [55].

The study will contribute to the evidence base for manual therapy management of headache and will lead to improved clinical decision making in the field of non-pharmacological management of headache.

## Data Availability

Data sharing is not applicable to this article as no datasets were generated or analysed during the current study.

## Abbreviations

TTH: Tension-type headache
CGH: Cervicogenic headache
ICHD: International Classification for Headache Disorders
MMT: Mulligan manual therapy
SNAG: Sustained natural apophyseal glide
FRT: Flexion Rotation Test
PPT: Pressure Pain Threshold
